# A new species of *Geotrigona* Moure from the Caribbean coast of Colombia (Hymenoptera, Apidae)

**DOI:** 10.3897/zookeys.172.2735

**Published:** 2012-03-01

**Authors:** Victor H. Gonzalez, Michael S. Engel

**Affiliations:** 1 Division of Entomology, Natural History Museum, and Department of Ecology & Evolutionary Biology, 1501 Crestline Drive – Suite 140, University of Kansas, Lawrence, Kansas 66045, USA

**Keywords:** Apoidea, Apinae, Anthophila, Joe Arroyo, Meliponini, Neotropics, stingless bees, taxonomy

## Abstract

A new species of the Neotropical stingless bee genus *Geotrigona* Moure from the Caribbean coast of Colombia is described and figured. *Geotrigona joearroyoi* **sp. n.** belongs to the *fulvohirta* species group and is distinguished on the basis of color and type of pubescence on the metasomal terga. New geographical records and an updated key to the species of *Geotrigona* are provided.

## Introduction

Stingless bees of the Neotropical genus *Geotrigona* Moure are robust, middle-sized (5–6.5 mm in body length), often black species superficially resembling some *Trigona* Jurine and *Partamona* Schwarz (Michener 2007). *Geotrigona* is widely distributed in the Neotropical region, occurring from Michoacán, Mexico to Santiago del Estero, Argentina, and inhabiting a diverse variety of ecosystems and climates, particularly along the Andes, where some species can be found at high altitudes. For example, *Geotrigona subrisea* (Cockerell) and *Geotrigona tellurica* Camargo & Moure have been collected at 3450 and 4000 m in the Colombian and Bolivian Andes; the latter altitude represents the highest elevation recorded for stingless bees in the Americas (Camargo and Moure 1996; Gonzalez and Engel 2004; Camargo and Pedro 2007; Gonzalez and Sepúlveda 2007). *Geotrigona* nest in the ground, hence their name, and the honey of some species is occasionally used by indigenous peoples, such as that of *Geotrigona acapulconis* (Strand) in Mexico (Camargo and Moure 1996; Michener 2007; Ayala et al. in prep.).

*Geotrigona* is related to *Trigona* and *Tetragona* Lepeletier de Saint Fargeau & Audinet-Serville, as suggested by both morphological and molecular analyses (Camargo and Moure 1996; Michener 2007; Rasmussen and Cameron 2010). The genus was revised by Camargo and Moure (1996), who recognized 16 species and several subspecies that were subsequently elevated to specific rank by Camargo and Pedro (2007), thus increasing the number to 20 species total; Gonzalez and Sepúlveda (2007) described an additional species from Colombia and revised the genus for the country. Based on a morphological phylogenetic analysis, Camargo and Moure (1996) recognized four species groups (Table 1) and discussed possible historical biogeographical scenarios. However, as mentioned by the authors, their phylogenetic hypotheses were not well-resolved, mainly because of the limited number of characters used, most of them recorded from the worker external morphology. Although meliponine taxonomy is based on workers, study of nesting behavior, males, and queens may provide additional characters useful in recognizing cryptic species and in phylogenetic analyses. Unfortunately, the nest and the male of *Geotrigona* are known for only about one-third of the species (Camargo and Moure 1996). Furthermore, despite being widely distributed in the Neotropical region, *Geotrigona* is rather poorly represented in biodiversity collections when compared to other stingless bee genera such as *Trigona* or *Partamona*; it is unknown from some countries where it is likely to occur based on the distribution (e.g., Venezuela, French Guiana). Without a doubt, a significant amount of work remains to be done in the systematics of the group. Herein, we describe a new species of *Geotrigona* of the *fulvohirta* species group based on workers collected in the Caribbean coast of Colombia, provide new geographical records, and an updated key to species of the genus.

**Table:** Table 1. Summary of currently included species in *Geotrigona* with information on the known sexes, nest and distribution. Sex/caste: ♀ = worker; ♂ = male; - = unknown. The distribution and nesting sites are based on Camargo and Moure (1996) and Camargo and Pedro (2007), with some modifications from Gonzalez and Sepúlveda (2007) and the present study.

Species	Sex	Nest	Distribution
“*fulvohirta* species group”			
*Geotrigona acapulconis* (Strand, 1919)	♀♂	+	Mexico
*Geotrigona chiriquiensis* (Schwarz, 1951)	♀	+	Panama
*Geotrigona fulvohirta* (Friese, 1900)	♀	+	Bolivia, Brazil, Colombia, Ecuador, Peru
*Geotrigona fumipennis* Camargo & Moure, 1996	♀	-	Ecuador
*Geotrigona joearroyoi* Gonzalez & Engel, sp. n.	♀	-	Colombia
*Geotrigona kaba* Gonzalez & Sepúlveda, 2007	♀		Colombia
*Geotrigona leucogastra* (Cockerell, 1914)	♀	-	Ecuador
*Geotrigona lutzi* Camargo & Moure, 1996	♀♂	-	Costa Rica, El Salvador, Guatemala, Honduras, Nicaragua
*Geotrigona terricola* Camargo & Moure, 1996	♀	-	Guatemala, Honduras
“*mombuca* species group”			
*Geotrigona aequinoctialis* (Ducke, 1925)	♀	+	Brazil
*Geotrigona argentina* Camargo & Moure, 1996	♀♂	+	Argentina, Bolivia, Paraguay
*Geotrigona fulvatra* Camargo & Moure, 1996	♀	-	Bolivia, Peru
*Geotrigona mattogrossensis* (Ducke, 1925)	♀♂	+	Brazil, Bolivia
*Geotrigona mombuca* (Smith, 1863)	♀♂	+	Brazil, Paraguay
*Geotrigona xanthopoda* Camargo & Moure, 1996	♀	-	Brazil
“*subterranea* species group”			
*Geotrigona subterranea* (Friese, 1901)	♀♂	+	Brazil
“*subgrisea* species group”			
*Geotrigona kraussi* (Schwarz, 1951)	♀	+	Panama
*Geotrigona kwyrakai* Camargo & Moure, 1996	♀	-	Brazil
*Geotrigona subfulva* Camargo & Moure, 1996	♀	-	Brazil, Colombia
*Geotrigona subgrisea* (Cockerell, 1920)	♀	-	Brazil, Colombia
*Geotrigona subnigra* (Schwarz, 1940)	♀	-	Brazil, Guyana
*Geotrigona tellurica* Camargo & Moure, 1996	♀♂	-	Bolivia, Ecuador, Peru

## Material and methods

Morphological terminology follows that of Michener (2007), except for torulus herein used instead of antennal alveolus or socket, while the format for the descriptions generally follows that used by Gonzalez and Sepúlveda (2007). Measurements were taken using an ocular micrometer on an Olympus SZX-12 stereomicroscope. Photomicrographs were taken using a Nikon D1x digital camera attached to an Infinity K-2 long-distance microscopic lens. Measurements in descriptions are for the holotype, with values for paratypes in parentheses. Institutional acronyms used herein are: **AMNH**, American Museum of Natural History, New York, USA; **ICN**, Instituto de Ciencias Naturales, Universidad Nacional de Colombia, Bogotá, Colombia; and **SEMC**, Snow Entomological Collection, Division of Entomology, University of Kansas Natural History Museum, Lawrence, Kansas, USA. The symbol for female and that word itself are used below for worker, not queen.

## Systematics

### Tribe Meliponini Lepeletier de Saint Fargeau, 1836. Genus Geotrigona Moure, 1943

#### Geotrigona joearroyoi sp. n.

urn:lsid:zoobank.org:act:14F5E52A-0EDD-4D51-BB44-8EA7CB2E65DD

http://species-id.net/wiki/Geotrigona_joearroyoi

Figs 1–5

##### Holotype.

 ♀, Colombia: Magdalena, Santa Marta, on the road from Bastidas to Bahía Concha, 11°15.874'N, 74°09.924'W; Dec 18, 2011, 99 m., V.H. Gonzalez (ICN).

##### Paratypes.

 Two workers with the same data as the holotype (SEMC, ICN).

##### Diagnosis.

**Figures 1–5.**
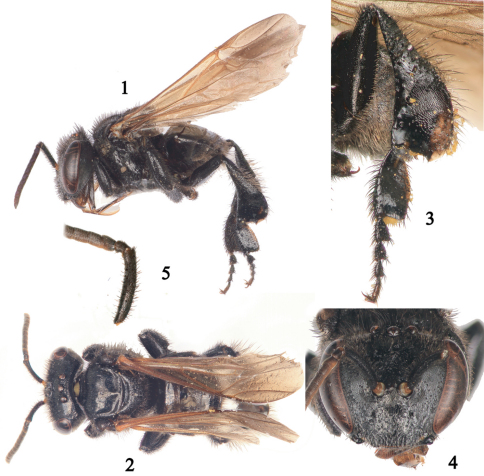
Worker of *Geotrigona joearroyoi* Gonzalez and Engel, sp. n. (holotype depicted except paratypes in figures 3, 5) **1** Lateral habitus **2** Dorsal habitus **3** Hind leg showing outer surfaces of metatibia and metabasitarsus **4** Facial view **5** Detail of antennal scape and basal flagellomeres.

 This species belongs to the *fulvohirta* species group *sensu* Camargo and Moure (1996) recognized by the metatibia with posterodistal margin distinctly projecting into an angle or tooth (Figs 1, 3). It is most similar to *Geotrigona fumipennis* Camargo & Moure sharing antennal scape with short setae (about as wide as half width of scape), body pubescence predominantly dark brown to black, and metabasitarsus with posterior margin slightly convex (Fig. 3). It can be distinguished from that species by metasomal terga with black setae except on sixth tergum (Figs 1, 2) and distal margins of second to fifth terga without appressed, branched setae. In *Geotrigona fumipennis* the metasomal terga have grayish setae and distal margins of second to fifth terga are distinctly covered by appressed, branched setae.

##### Description.

*Worker*: Total body length 5.2 mm (4.8–5.2 mm); head width 2.5 mm (2.4–2.5 mm); forewing length (measured from apex of humeral sclerite) 5.6 mm (5.6–5.7 mm). Head 1.3 times wider than long; inner orbits of compound eyes converging below (Fig. 4); malar area short, about 0.4 times width of third flagellomere; clypeus about 1.7 times broader than long; intertorular distance about as long as torular diameter; torulorbital distance about twice as long as torular diameter; interocellar distance 2.3 times median ocellar diameter, 1.1 times longer than ocellocular distance; ocelloccipital distance about half median ocellar diameter; scape 6 times longer than wide, about as wide as width of third flagellomere; pedicel about as long as broad, shorter than first flagellomere; flagellomeres slightly longer than broad, apical flagellomere longest; compound eye 2.9 times longer than broad; gena about as broad as compound eye in profile. Metatibia 2.7 times longer than broad with posterodistal margin distinctly projected into an angle, distal margin emarginate between projection and penicillum, corbicula on distal one-third; metabasitarsus about twice as long as broad, slightly convex on posterior margin (Fig. 3).

Integument smooth and shiny, as in other species of the genus.

Color black, including tegula and humeral sclerite, except dark reddish brown on mandible distally, flagellum (yellowish ventrally), and distitarsi. Wing membranes and veins light ferruginous, slightly dusky distally including pterostigma (Figs 1, 2).

Body pubescence black, except: inferior half of face and gena with dense, branched, short, appressed grayish setae; superior half of face, pronotal lobe, metepisternum, sides of propodeum, and metatibia basally with dark brown setae; tergum six and sterna with grayish setae. Clypeus with erect setae about 0.6 times median ocellar diameter; scape with abundant, short, erect, simple setae, about as long as or slightly longer (0.5–0.6 times) than half width of scape (Fig. 5); frons with longer erect setae than on clypeus, 1.2 times median ocellar diameter; vertex with erect setae 1.6–1.8 times median ocellar diameter; mesoscutum with erect setae about as long as median ocellar diameter, longer on anterior margin; mesoscutellum and mesepisternum with erect setae 1.2 times median ocellar diameter; metatibia with long erect setae, 2.4–2.8 times median ocellar diameter. First metasomal tergum practically glabrous, with scattered, minute erect setae on disc and denser, longer (0.5 times median ocellar diameter) erect setae laterally; remaining terga with simple, semierect to erect setae progressively increasing in density and length towards distal terga; tergum sixth with both simple and appressed, branched setae (1.2–1.6 times median ocellar diameter).

*Male*: Unknown.

*Queen*: Unknown.

##### Etymology.

 The species is named in tribute to the late Colombian tropical music singer, composer, and songwriter Alvaro José Arroyo González (1 November 1955–26 July 2011). This artist, also known as Joe Arroyo or El Joe, was nationally and internationally known for his unique way of combining a diverse array of Caribbean music styles, including salsa, cumbia, porro, soca, kompa, and zouk (Harris 2012).

##### Comments.

 Based on the limited material available it appears that *Geotrigona joearroyoi* and *Geotrigona fumipennis* are allopatric species: *Geotrigona joearroyoi* inhabiting lowland dry forests in the Colombian Caribbean, *Geotrigona fumipennis* occupying lowland dry forests as well as montane to premontane rain forests along the western slope of the Andes in southern Ecuador (Camargo and Moure 1996).

#### Geotrigona fulvohirta

(Friese)

http://species-id.net/wiki/Geotrigona_fulvohirta

Trigona fulvohirta Friese, 1900: 385 [♀]

##### New record.

Colombia: 4♀♀, Meta, Villavicencio [4°08'N, 73°40'W; 467 m], B. Colina; M. Salazar, E. Palacios. 20-04-04 [April 20, 2004] (ICN).

##### Comments.

 This species was previously known in Colombia from the departments of Amazonas, Boyacá, and Putumayo (Camargo and Pedro 2007; Gonzalez and Sepúlveda 2007).

#### Geotrigona kaba

Gonzalez & Sepúlveda

http://species-id.net/wiki/Geotrigona_kaba

Geotrigona kaba Gonzalez & Sepúlveda, 2007: 104 [♀]

##### New record.

Colombia: 1♀, Dept. Boyacá, Muzo [5°31'48"N, 74°6'36"W], 900 m, 1936, J. Bequaert collector (AMNH).

##### Comments.

 This species was previously known only from the type locality (Porce, Antioquia) in northwestern Colombia. The worker at the AMNH was collected on the western slope of the Eastern Andes in central Colombia and bears a red label indicating a holotype designation by H.F. Schwarz, who intended to name it after the department.

#### Geotrigona mombuca

(Smith)

http://species-id.net/wiki/Geotrigona_mombuca

Trigona mombuca Smith, 1863: 509 [♀]
Geotrigona inusitata Moure and Camargo, 1992: 53 [♀]

##### New record.

Paraguay: 1♀, Alto Paraguay: Parque Nacional Defensores del Chaco, Cruce 4 de Mayo, Mojón 16, 18.i.2001, B. Garcete, coll. (SEMC).

##### Comments.

 This species was previously known in Paraguay from the state of Misiones (Camargo and Pedro 2008).

#### Geotrigona subgrisea

(Cockerell)

http://species-id.net/wiki/Geotrigona_subgrisea

Trigona subgrisea Cockerell, 1920: 465 [♀]

##### New record.

Colombia: 1♀, Huila, San Agustín, Hostal Huaka-Yo, 1°53.311'N, 76°17.812W, 1748 m; Dec 29, 2011; V.H. Gonzalez (SEMC).

##### Comments.

 This species is known from Brazil (Roraima) and Colombia (Departaments of Boyacá, Cundinamarca, Putumayo, and Tolima). Camargo and Moure (1996) examined a single female specimen from Tolima, which had a low carina on the vertex, in comparison to the Brazilian specimens; they suspected of a differentiated population of this species in Colombia. Gonzalez and Sepúlveda (2007) expanded the distribution range in Colombia, noting that all records came from the Andean region (as in this new record) as well as minor differences in the wing color, namely the forewing dusky apically. It is likely that the Colombian specimens are actually a distinct species given these subtle differences but more importantly their allopatric distribution; however, more records as well as the study of males of both species are necessary to test this hypothesis.

### Key to species of Geotrigona (workers)

Modified from Camargo and Moure (1996).

**Table:** 

1	Metatibia with posterodistal margin broadly rounded, not projecting into a distinct angle or tooth; distal margin straight or weakly emarginate	2
–	Metatibia with posterodistal margin distinctly projecting into an angle or tooth (Fig. 3); distal margin, between tooth and penicillum, strongly emarginate (*fulvohirta* species group)	14
2(1)	Vertex with distinct carina behind ocelli (*subgrisea* species group)	3
–	Vertex slightly elevated or rounded, without distinct carina behind ocelli (*mombuca* species group)	8
3(2)	Vertex and mesoscutum predominantly with dark brown setae	4
–	Vertex and mesosoma predominantly with light ferruginous setae	5
4(3)	Vertex with low (~ 0.06 mm in height), somewhat vertical carina (Panama)	*Geotrigona kraussi* (Schwarz)
–	Vertex with higher (0.08–0.10 mm), anteriorly directed carina, nearly covering posterior margin of lateral ocelli (Brazil, Guyana)	*Geotrigona subnigra* (Schwarz)
5(3)	Wing membranes uniformly light ferruginous; scape with setae shorter than half width of scape (eastern slope of Andean region of Bolivia, Peru, and Ecuador)	*Geotrigona tellurica* Camargo & Moure
–	Wing membranes hyaline; scape with setae of variable length among species	6
6(5)	Scape with setae shorter than half width of scape; legs with light ferruginous setae as on remaining areas of body (Brazil, Colombia)	*Geotrigona subgrisea* (Cockerell)
–	Scape with longer setae, at least half width of scape, distinctly branched apically; legs with pubescence of variable color among species	7
7(6)	Legs with light ferruginous setae as on remaining areas of body (Brazil: Paraná, Rondônia)	*Geotrigona kwyrakai* Camargo & Moure
–	Legs with brownish-grey setae contrasting with light reddish brown setae on remaining areas of the body (Brazil: Amazonas; Colombia: Amazonas)	*Geotrigona subfulva* Camargo & Moure
8(2)	Metatibia yellowish contrasting with black integument on remaining areas of body; body with dark brown to black setae except on sterna and apical terga with whitish setae	*Geotrigona xanthopoda* Camargo & Moure
–	Metatibia dark brown to black as on remaining areas of body; setae of variable color among species	9
9(8)	Body pubescence light ferruginous	10
–	Body pubescence either predominantly whitish or black or a mixture of both	11
10(9)	Interocellar distance slightly longer than ocellocular distance; wing membranes light ferruginous basally, dusky apically	*Geotrigona fulvatra* Camargo & Moure
–	Interocellar distance distinctly longer than ocellocular distance (such a difference equal to half median ocellar diameter); wing membranes subhyaline, not bicolorous as above	*Geotrigona mattogrossensis* (Ducke)
11(9)	Mesepisternum predominantly with whitish setae	12
–	Mesepisternum predominantly with dark brown to black setae	13
12(11)	Wing membranes hyaline (Paraguay; northeastern, central west, and southeastern Brazil)	*Geotrigona mombuca* (Smith)
–	Wing membrane slightly ferruginous (Brazil: Pará, Maranhão, Ceará)	*Geotrigona aequinoctialis* (Ducke)
13(11)	Small bees (head width: ≤ 2.4 mm); vertex with short (less than one-fourth length of scape) and thick setae; scape with setae at most one-third width of scape; metasomal sterna, especially basal segments, with brownish-grey setae; wing membranes slightly brownish	*Geotrigona argentina* Camargo & Moure
–	Larger bees (head width: 2.6–2.7 mm); vertex with longer (about two-fifths length of scape) and thinner setae; scape with abundant and longer setae, about two-fifths width of scape; metasomal sterna grayish setae; wing membranes variable, hyaline or subhyaline to slightly brownish	*Geotrigona subterranea* (Friese)
14(1)	Scape with long setae, at least 0.75 times width of scape	15
–	Scape with short setae, at most half width of scape (Fig. 5)	19
15(14)	Head (including scape) and mesosoma with predominantly light ferruginous setae	16
–	Head and mesosoma with predominantly dark brown setae	17
16(15)	Scape with long setae, nearly twice as long as width of scape; metasoma with predominantly light ferruginous setae; wings membrane subhyaline, slightly yellowish (Panama)	*Geotrigona chiriquiensis* (Schwarz)
–	Scape with shorter setae, about 1.4 times width of scape; metasoma with predominantly whitish or grayish setae; wings membrane hyaline, darker apically (Colombia)	*Geotrigona kaba* Gonzalez & Sepúlveda
17(15)	Scape with short setae, about 0.75 times width of scape	*Geotrigona terricola* Camargo & Moure
–	Scape with longer setae, 1.4–1.5 times width of scape	18
18(17)	Wing membranes hyaline, veins and microtrichia honey colored (Ecuador, Pacific coast)	*Geotrigona leucogastra* (Cockerell)
–	Wing membranes light ferruginous, slightly darkened, veins and microtrichia dark brown (Guatemala to Costa Rica)	*Geotrigona lutzi* Camargo & Moure
19(14)	Body pubescence predominantly light ferruginous; forewing light ferruginous basally, darker distally, particularly marginal cell	*Geotrigona fulvohirta* (Friese)
–	Body pubescence predominantly brownish-grey or black; forewing entirely light ferruginous, not bicolorous as above	20
20(19)	Scape and mesepisternum with whitish setae; metabasitarsus with posterior margin straight or nearly so (Mexico)	*Geotrigona acapulconis* (Strand)
–	Scape and mesepisternum with dark brown to black setae; metabasitarsus with posterior margin slightly convex (Ecuador, Colombia)	21
21(20)	Metasomal terga with grayish setae, third to sixth terga distally with distinct, appressed, branched setae (western Ecuador)	*Geotrigona fumipennis* Camargo & Moure
–	Metasomal terga with black to dark brown setae except grayish on tergum sixth, second to fifth terga distally without appressed, branched setae, present only on sixth tergum (Colombian Caribbean)	*Geotrigona joearroyoi* sp. n.

## Supplementary Material

XML Treatment for Geotrigona joearroyoi

XML Treatment for Geotrigona fulvohirta

XML Treatment for Geotrigona kaba

XML Treatment for Geotrigona mombuca

XML Treatment for Geotrigona subgrisea
